# Microscopic biopsy after unsuccessful endoscopic biopsy for primary central nervous system lymphoma of the corpus callosum: A case report

**DOI:** 10.1016/j.amsu.2021.102746

**Published:** 2021-08-24

**Authors:** Tokunori Kanazawa, Kosuke Karatsu, Takumi Kuramae, Masayuki Ishihara

**Affiliations:** Department of Neurosurgery, National Hospital Organization Tochigi Medical Center, 1-10-37, Nakatomatsuri, Utsunomiya, Tochigi, Japan

**Keywords:** PCNSL, Endoscopic biopsy, Microscopic biopsy, 5-ALA, Neuronavigation

## Abstract

**Introduction and importance:**

Primary central nervous system lymphoma (PCNSL) is a rare tumor with a poor prognosis. Early brain biopsy is essential to avoid a diagnostic delay. To date, reports of successful diagnosis for PCNSL of the corpus callosum by endoscopic biopsy are rare.

**Case presentation:**

Herein, we report the case of an elderly woman with PCNSL of the corpus callosum who initially presented with rapidly progressive dementia. The condition was finally diagnosed by microscopic biopsy after unsuccessful endoscopic biopsy. Moreover, the postoperative course was uneventful. She is currently receiving systemic chemotherapy.

**Clinical discussion:**

Early diagnosis and subsequent systemic chemotherapy with or without whole brain radiotherapy are critical for PCNSL. Endoscopic biopsy may be a diagnostic option for suspected PCNSL, although stereotactic needle biopsy is most commonly used.

**Conclusion:**

Utilizing neuronavigation and 5-aminolevulinic acid (ALA) fluorescence guidance could be helpful in identifying lesions insufficiently exposed by endoscopic visualization. However, cerebrospinal fluid (CSF) loss due to the endoscopic approach through the ventricle might be a cause of neuronavigation misregistration.

## Highlights

• Reports of the successful diagnosis of PCNSL by endoscopic biopsy are rare.
• However, endoscopic biopsy may be a diagnostic choice for suspected PCNSL.
• Neuronavigation and 5-ALA fluorescence guidance could be helpful.
• CSF loss may be a cause of neuronavigation misregistration as with this case.

## 1 Introduction

Primary central nervous system lymphoma (PCNSL) is a malignant and an aggressive non-Hodgkin lymphoma with a poor prognosis, confined to the central nervous system. It is a rare tumor, accounting for 2–4% of all brain tumors [1,2]. Regardless of recent advances in neuroimaging, radiographic imaging findings are suggestive but not diagnostic of PCNSL; therefore, a definitive diagnosis must be made by histopathological confirmation via brain biopsy. Early brain biopsy has been recommended to prevent a diagnostic delay, improve survival, and reduce neurological deficits. Although stereotactic or open biopsy is commonly performed, stereotactic biopsy is the most common diagnostic procedure for patients with suspected PCNSL [1,3], since most lesions are located deep into the brain. However, to the best of our knowledge, only a few cases of PCNSL diagnosis by endoscopic biopsy have been reported in the literature [[4], [5], [6]]. Therefore, we report a unique case of PCNSL of the corpus callosum presenting with rapidly progressive dementia, successfully diagnosed by microscopic biopsy after unsuccessful endoscopic biopsy, along with potential pitfalls in diagnosing PCNSL. This work is reported in accordance with SCARE Criteria [7].

## 2 Case presentation

**Fig. 1:**
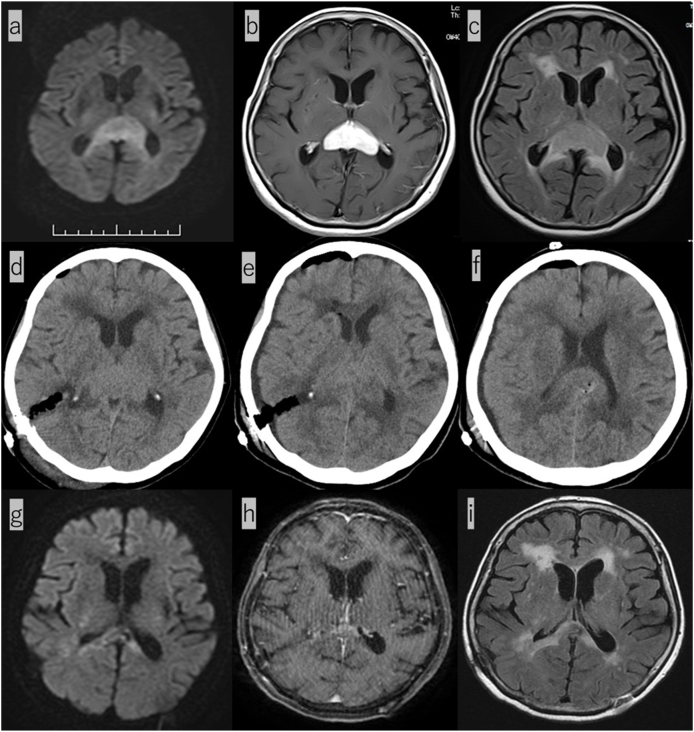
(a–c); a: DWI, b: contrast-enhanced T1, c: FLAIR) Initial head MRI reveals a contrast-enhancing lesion on the corpus callosum in the splenium with perifocal edema. (d) Postoperative CT after the 1st endoscopic biopsy shows the endoscopic approach via the trigon of the lateral ventricle. (e, f) Postoperative CT after the 2nd microscopic biopsy reveals an appropriate enucleation of the tumor. (g–i; g: DWI, h: contrast-enhanced T1, i: FLAIR) After 5 cycles of chemotherapy, head MRI reveals a reduction in the tumor size.

**Fig. 2:**
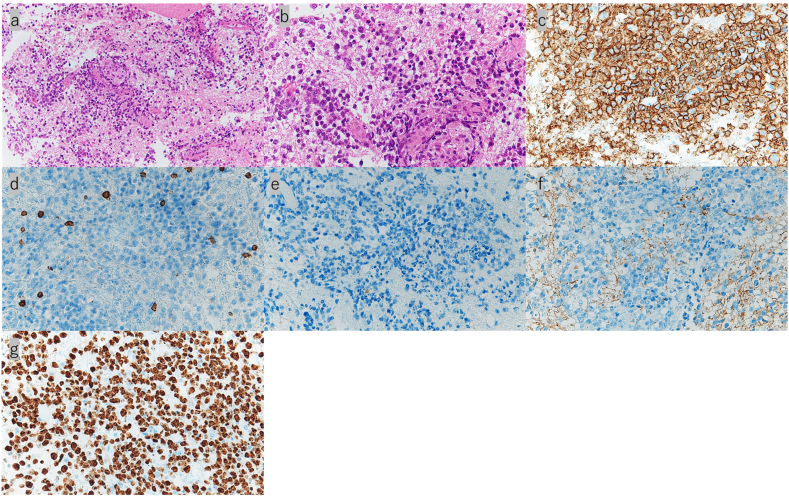
(a, b) Microscopic sections of the tumor show findings consistent with DLBCL. (c–f) Immunohistochemical studies show strong positivity forCD 20 and negativity for CD 3, cytokeratin AE1/AE3, and GFAP. (g) The cell proliferation index (Ki-67) was approximately 90%. (a, b: hematoxylin and eosin stain; a: × 100 magnified, b: × 400 magnified; c–g: immunostaining; c–g: × 400 magnified).

A 73-year-old woman presented to our unit via ambulance with rapid cognitive decline. Her medical history included hypertension and breast cancer. Clinical examination revealed rapidly progressive dementia (mini-mental status examination = 15/30), with no limb paralysis. A routine laboratory examination revealed normal IL-2 receptor (313 U/mL) andβ-2 MG (1.6 mg/L), without any other abnormalities. Head magnetic resonance imaging (MRI) showed a contrast-enhancing lesion on the corpus callosum in the splenium with perifocal edema (Fig. 1 a, b, c). Therefore, due to the suspicion of a glioma or PCNSL, endoscopic biopsy in conjunction with a neuronavigation system was performed through a parieto-occipital approach into the trigone of the lateral ventricle (Fig. 1 d). This surgery failed to achieve a definitive histologic diagnosis due to poor visualization. Thereafter, additional neuronavigation-guided microscopic biopsy was performed (Fig. 1 e). Her postoperative computed tomography (CT) revealed an appropriate enucleation of the tumor (Fig. 1 f). She had no new neurological deficits postoperatively. Microscopically, the lesion comprised neoplastic proliferation of lymphoid cells along perivascular spaces with a high N/C ratio, large vesicular nuclei, and high mitotic figures (Fig. 2 a, b). Immunohistochemical staining was positive for CD 20 (Fig. 2 c) and negative for CD 3 (Fig. 2 d), cytokeratin AE1/AE3 (Fig. 2 e), and glial fibrillary acidic protein (GFAP) (Fig. 2 f), confirming a high-grade diffuse large B-cell lymphoma (DLBCL). The Ki-67 labeling index was approximately 90% (Fig. 2 g). The check-up to rule out systemic lymphoma by whole-body CT scan revealed no apparent lesions. Ophthalmologic examination also indicated no ocular involvements. Based on these results, the patient was finally diagnosed with PCNSL. She was treated with an induction rituximab, methotrexate, procarbazine, and vincristine (R-MPV) chemotherapy (1 cycle = 14 days). After 5 cycles of chemotherapy, her head MRI revealed a reduction in the tumor volume (Fig. 1 g, h, i). Three months after the admission, she was discharged with a modified Rankin Scale score of 3. She has been followed closely without additional treatment.

## 3 Discussion

To date, there have been only a few reports of the successful diagnosis of PCNSL of the corpus callosum by endoscopic biopsy [[4], [5], [6]]; therefore, we report a rare case of PCNSL of the corpus callosum successfully diagnosed by additional microscopic biopsy after an unsuccessful endoscopic biopsy.

PCNSL is an uncommon neurosurgical presentation in daily practice; however, a diagnostic delay can lead to a poor prognosis [6]. Therefore, early diagnosis is critical for patients with PCNSL, since histopathologic diagnosis is indispensable in planning appropriate treatment including systemic chemotherapy with or without whole brain radiotherapy. Unless cerebrospinal fluid (CSF) or vitreoretinal cytopathology and flow cytometry are diagnostic, the common diagnostic procedure is brain biopsy [1,3], such as stereotactic needle, open microscopic, and endoscopic biopsies. Unlike other brain tumors, previous reports recommend not to attempt to perform PCNSL resection [8,9], since the extent of resection has no prognostic impact. Among them, the diagnosis is most often obtained via stereotactic needle biopsy; however, the choice of the surgical approach for suspected PCNSL depends on the surgeon's preference and experience. The National Comprehensive Cancer Network guidelines suggest biopsy using the least invasive approach.

In our case, endoscopic biopsy was initially selected as the minimally invasive treatment, but required an additional surgery to achieve a definitive histologic diagnosis, since we could not reach the appropriate lesion due to poor endoscopic visualization. The use of 5-aminolevulinic acid (5-ALA) might contribute to safe endoscopic biopsy for accurate diagnosis, since fluorescence-guided endoscopic visualization identified 5-ALA-positive tissue not sufficiently exposed by conventional microscopic visualization as previously reported [10]. Endoscopic biopsy was performed under neuronavigation guidance to compensate for poor visualization in endoscopy, but the guidance was insufficient to perform an accurate biopsy. This difficulty might be attributed to the positional displacement of neuronavigation due to CSF loss via the trigon of the lateral ventricle as a result of the endoscopic approach. Furthermore, microscopic visualization was finally better than the endoscopic one, which resulted in the definitive diagnosis in our case.

## 4 Conclusion

In conclusion, PCNSL is a rare presentation, but a diagnostic delay can lead to a poor prognosis. Early diagnosis by brain biopsy and subsequent systemic chemotherapy with or without whole brain radiotherapy are of critical importance. Endoscopic biopsy may be a diagnostic option for suspected PCNSL. The use of neuronavigation and 5-ALA fluorescence guidance could contribute to identify lesions insufficiently exposed by endoscopic visualization. However, CSF loss due to the endoscopic approach through the ventricle might lead to the misregistration of neuronavigation like our case.

## Consent

Written informed consent was obtained from the patient for publication of this case report and accompanying images. A copy of the written consent is available for review by the Editor-in-Chief of this journal on request.

## Ethical approval

Since this is a case report, ethical approval was not required from Institutional Review Board.

## Sources of funding

The authors received no specific funding for this work.

## Author contribution

Study concept or design: Tokunori Kanazawa.
Data collection: Tokunori Kanazawa.
Data analysis or interpretation: Tokunori Kanazawa.
Writing the paper: Tokunori Kanazawa.
Management of this patient: Kosuke Karatsu and Tokunori Kanazawa.
Supervisor: Takumi Kuramae and Masayuki Ishihara.

## Research registration

This is a case report so registration was not required.

## Guarantor

All the authors are the guarantor of the study.

Provenance and peer review.

Not commissioned, externally peer-reviewed.

## Declaration of competing interest

The authors declare that they have no conflict of interest.
